# The evaluation of main sources of anxiety and fear for Covid 19 in Greece

**DOI:** 10.1192/j.eurpsy.2022.1332

**Published:** 2022-09-01

**Authors:** G. Lyrakos, G. Pilafas, E. Aslani, V. Spinaris

**Affiliations:** 1 City Unity College, Psychology, Athens, Greece; 2 General Hospital of Nikaia, Psychiatric, Nikaia, Greece; 3 General Hospital Nikaia Ag. Panteleimon, 2nd Covid Clinic, Nikaia, Greece

**Keywords:** fear, Covid-19, Anxiety

## Abstract

**Introduction:**

Covid19 has led in major changes in our lives, while fear was one of the major psychological symptoms that emerged in psychological first aid evaluations.

**Objectives:**

The aim of the present study was to report the major factors creating anxiety and fear, affecting everyday life of people in Greece during the two years of the pandemic.

**Methods:**

A sample of 1,158 Greeks (280 males [24.2%] participated voluntarily in the study through online platforms. The Fear factors was assessed through an open question which was then analyzed with SPSS 24.

**Results:**

According to the findings, the main source of anxiety and fear arises from the situation that prevails in other European countries with increasing death rates (20.9%) presented in television, followed by what is shown in the news and news programs on television (14, 8%), the fear that the individual may get sick, watching television and radio (9.7%), the experts announcements in public (7.8%), the announcement of new measures by the government and the Ministry of Health in media (6.1%), the existence of elderly parents in the family (4.6%) and social networks (3.8%), while 12.6% stated that they have no fear or anxiety. Gender differences were significant in most of the factors x^2^=51.167 p=.001.

**Conclusions:**

According to the findings the effect that media have in anxiety and fear creation (64.1%), a result that can be used in designing effective health measures that can help people deal with the psychological aftermath of the pandemic.

**Disclosure:**

No significant relationships.

